# Optimising test intervals for individuals with type 2 diabetes: A machine learning approach

**DOI:** 10.1371/journal.pone.0317722

**Published:** 2025-02-13

**Authors:** Sasja Maria Pedersen, Nicolai Damslund, Trine Kjær, Kim Rose Olsen

**Affiliations:** DaCHE, Department of Public Health, University of Southern Denmark, Odense, Denmark; Universita Politecnica delle Marche, ITALY

## Abstract

**Background:**

Chronic disease monitoring programs often adopt a one-size-fits-all approach that does not consider variation in need, potentially leading to excessive or insufficient support for patients at different risk levels. Machine learning (ML) developments offer new opportunities for personalised medicine in clinical practice.

**Objective:**

To demonstrate the potential of ML to guide resource allocation and tailored disease management, this study aims to predict the optimal testing interval for monitoring blood glucose (HbA1c) for patients with Type 2 Diabetes (T2D). We examine fairness across income and education levels and evaluate the risk of false-positives and false-negatives.

**Data:**

Danish administrative registers are linked with national clinical databases. Our population consists of all T2D patients from 2015-2018, a sample of more than 57,000. Data contains patient-level clinical measures, healthcare utilisation, medicine, and socio-demographics.

**Methods:**

We classify HbA1c test intervals into four categories (3, 6, 9, and 12 months) using three classification algorithms: logistic regression, random forest, and extreme gradient boosting (XGBoost). Feature importance is assessed with SHAP model explanations on the best-performing model, which was XGBoost. A training set comprising 80% of the data is used to predict optimal test intervals, with 20% reserved for testing. Cross-validation is employed to enhance the model’s reliability and reduce overfitting. Model performance is evaluated using ROC-AUC, and optimal intervals are determined based on a “time-to-next-positive-test” concept, with different durations associated with specific intervals.

**Results:**

The model exhibits varying predictive accuracy, with AUC scores ranging from 0.53 to 0.89 across different test intervals. We find significant potential to free resources by prolonging the test interval for well-controlled patients. The fairness metric suggests models perform well in terms of equality. There is a sizeable risk of false negatives (predicting longer intervals than optimal), which requires attention.

**Conclusions:**

We demonstrate the potential to use ML in personalised diabetes management by assisting physicians in categorising patients by testing frequencies. Clinical validation on diverse patient populations is needed to assess the model’s performance in real-world settings.

## Introduction

Access to big data, including machine learning (ML) applications for predictions, has been advocated as a prospect for improved diagnostics and treatment decisions, enhancing personalised medicine. This has led to a rapid increase in the number of published ML studies in the area of health [1–3]. While this is promising, most ML studies tend to focus on predictive performance as their endpoint, often paying less attention to key results and biases that are relevant from an implementation perspective. ML algorithms have been proposed as a means to eliminate subjective human biases in clinical decision-making. However, the premise that ML algorithms are more objective and fair than human decision-makers largely depends on the data influx used to train them. It is important to acknowledge that administrative health data are behavioural since they rely on data on healthcare utilisation among those who seek healthcare, rather than the actual presence of disease, or symptoms. Such selection problems may result in algorithms that, if implemented, may preserve historical inequalities, and even augment them in future decision-making processes [4, 5]. To assess and minimise this bias, fairness metrics have been suggested as part of the model evaluation process [6]. Fairness entails that prediction models treat all individuals equally, regardless of their underlying characteristics, such as socio-economic status. Moreover, for models to prove successful, it is paramount that healthcare professionals and decision-makers understand the results and have confidence in the predictions [7, 8]. This includes information on feature importance and the risk of false positives (specificity) and false negatives (sensitivity). Feature importance provides valuable insight into the relationship between input features and model predictions. It is a crucial measure to improve accountability in the decision-making process and guide feature selection [9]. In addition, the rate of false positives and false negative have implications on the model’s applicability in clinical settings [10]. Often, ML models involve the predicted classification of patients into different treatment paths based on needs, allowing for the reallocation of healthcare with allocation of more resources to high-need patients and fewer resources to low-need patients. In such cases, the risk of wrong classification of patients may dissuade healthcare professionals from trusting ML algorithms to guide individualised healthcare as they may be concerned about the potential harm to the patients classified as low-need.

Our study adds to the small strand of literature using ML predictions to guide resource allocation in public health [11, 12]. Specifically, we aim to illustrate the potential of using ML on administrative data to facilitate personalised medicine in healthcare delivery focusing on results and potential biases that are key from an implementation perspective. We define personalised medicine in line with the previous literature, as patient-tailored healthcare that optimises the chances of receiving the proper treatment at the right dose and at the right time [13]. Several studies in the area of health have used ML to predict the development of diabetes, diabetes complications and HbA1c trajectories [14–17]. Our study contributes to the literature by predicting optimal intervals for HbA1c testing in general practice, based on individual characteristics. We utilise data on glycemic status (HbA1c) for individuals with type 2 diabetes (T2D) in Denmark. Monitoring HbA1c is important as elevated levels (above 48 mmol/mol) are known to increase the risk of complications. Our study is motivated by a high incidence and increasing prevalence of diabetes implying extensive resource use on even simple tasks as HbA1c measurement. Furthermore, we observe that nearly 40% of all HbA1c tests conducted among T2D individuals are below the critical threshold of 48 mmol/mol, suggesting that testing intervals safely could be extended for these individuals. Adapting to an increasing diabetes prevalence is crucial for healthcare providers when resources are constrained. This observation raises questions about the current testing time intervals. Based on clinical evidence, we expect that HbA1c test results serve as a reliable predictor for the ideal time gap between tests for an individual [18]. The stability and dependability of HbA1c measurements further establish its efficacy as a predictive factor in determining the optimal testing frequency for an individual.

We expect the predictive performance to increase further using readily available retrospective administrative and laboratory data [19]. The optimal test interval is defined as the predicted interval to a HbA1c measurement above the clinical threshold for well-controlled patients. While the current guidelines recommend testing every 3 to 6 months, we expand the prediction to include 9-month and 12 month intervals. This enables prediction of longer optimal intervals for patients with expected stable glycemic control. Consequently, reducing testing intervals (in these cases), will free resources for patients who struggle to manage their glycemic control and thus require more frequent testing.

We employ three algorithms: Logistic regression, Random Forest and the Extreme Gradient Boosting (XGBoost) ML algorithm to predict the optimal test interval for HbA1c. We approach the problem as a multiclass classification task with four test intervals (3, 6, 9 and 12 months) as potential outcomes. To examine the feature importance of predicted optimal interval, we use Shapely Additive Explanations (SHAP model explanations) [20]. Moreover, we investigate the fairness in the ML model by income and education and analyse the risk of bias in the classifications of the optimal interval in terms of false-positive and false-negative rates.

## Methods

### Monitoring of the diabetes population

**Fig 1:**
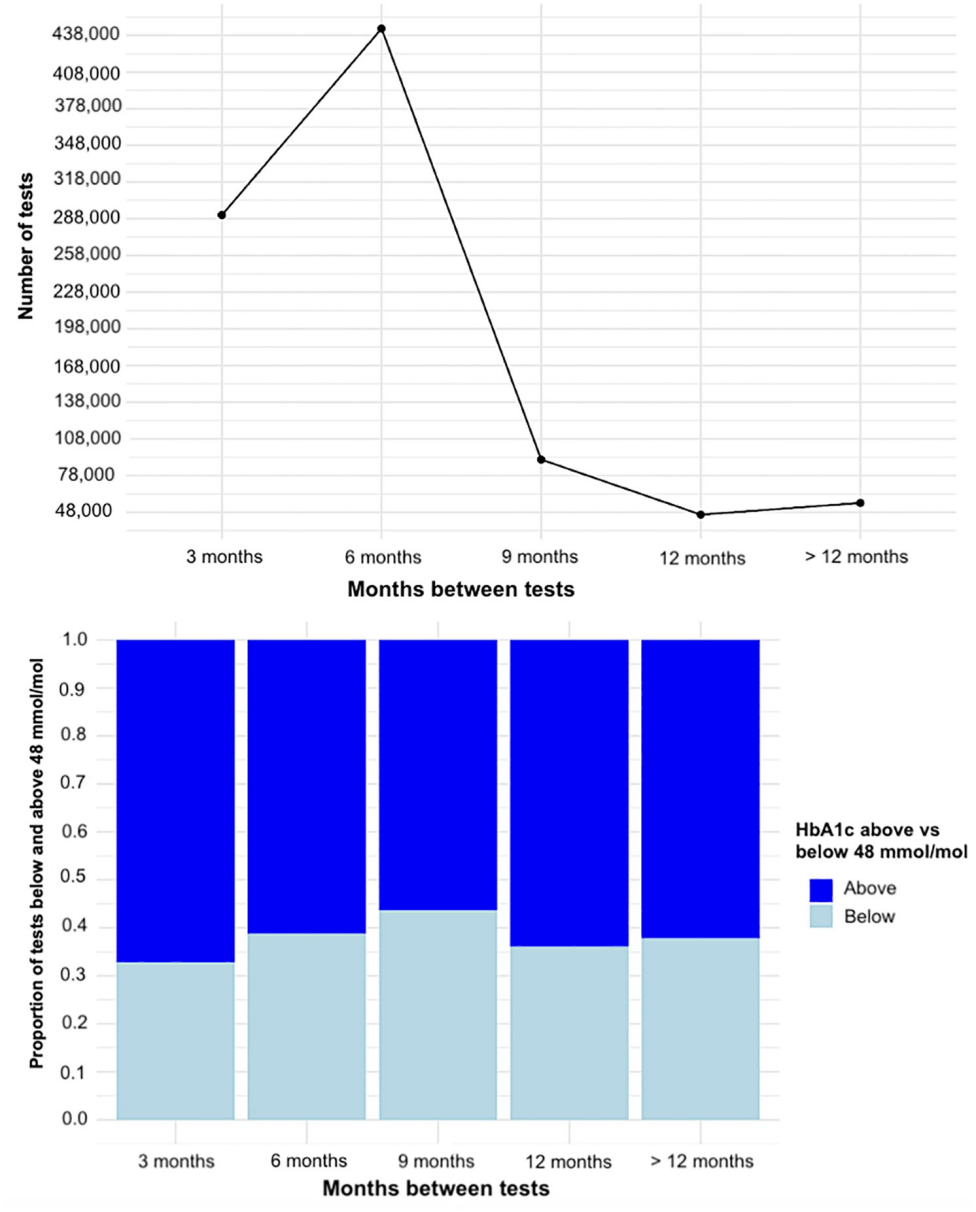
Overall Danish diabetes population. A: Months between tests and proportion of tests below and above 48 mmol/mol. B: Months between tests and number of tests.

In Denmark, general practitioners (GPs) are responsible for the managing of patients with T2D. As illustrated in Fig 1A, most T2D patients are tested within an interval of 6 months or less, in line with current guidelines, whereas very few tests occur within a longer interval of 9–12 months. Notably, around 35% of tests conducted at intervals between 3–6 months show an HbA1c level below 48 mmol/mol. This share is even higher (41%) for tests conducted at 6–9-month intervals, suggesting potential for implementing more individualised testing intervals (Fig 1B).

### Study population

The study population consists of a cohort of people with T2D as of the 1st of January 2015. The cohort is followed until 2018. The definition of T2D is based on the RUKS algorithm used by the Danish Health Data Authority [21]. RUKS is based on ICD10 codes and prescribed pharmaceuticals. As a further inclusion criteria, we only include individuals with at least one HbA1c test every year (+ 3 months) and individuals under the age of 70. We do this to limit our sample to individuals regularly monitored by their GP and complying with the national guidelines for T2D monitoring [22]. In this way we seek to match our model to the pool of patients that would be eligible for entering a ML guided monitoring programme. As the current guidelines recommend testing every 3 to 6 months, we have three groups in our data. One group of patients where some individuals are likely to receive more services than needed given the current provision of healthcare (over provision of services). A second group of individuals with a risk of having unmet need (under provision of services), and a third group of individuals receiving adequate provision of healthcare. Due to the lack of national administrative records of HbA1c, our population is limited to include individuals from four of the five regions in Denmark. The Region of Central Jutland is excluded as HbA1c test results are not reported to the national registers. This gives us a final sample of 57,872 individuals, comprising 405,616 HbA1c test observations over the period 2015–2018.

### Data

We use data from the comprehensive collection of Danish administrative registers from 2015–2018. Data across registers are collated by social security number, a unique number assigned to all individuals living in Denmark. As is standard in the ML literature, we use the term ‘features’ for variables used to predict the outcome. In selecting features for our model, we choose features that are related to diabetes management, including demographic variables, blood test results relevant to diabetes, health information (diagnoses and prescription medicine), and general practitioner (GP) utilisation. See Table 1 for an overview of the data and selected features.

**Table 1:** Predictor variables.

*Data segments and variables*	*Databases*						
	Lab^1^	CPR^2^	IND^3^	UDDA^4^	LPR^5^	LMDB^6^	SSR^7^
**1. Demographics**							
Sex		✔					
Age		✔					
Civil status		✔					
Family income			✔				
Education				✔			
Occupation			✔				
**2. Laboratory**							
Triglyceride	✔						
Total Cholesterol	✔						
HDL Cholesterol	✔						
LDL Cholesterol	✔						
Creatinine	✔						
HbA1c	✔						
**3. Health**							
Charlson Index (CCI) ^a^					✔		
ICD10 diagnosis					✔		
Medication utilization—ATC codes						✔	
**4. GP utilization**							
GP costs							✔
Tests at the GP							✔
GP contacts							✔

^1^ LAB: Laboratory data

^2^ CPR: Population data

^3^ IND: Income data

^4^ UDDA: Education data

^5^ LPR: Hospitalization data

^6^ LMDB: Prescription’s data

^7^ SSR: Primary care

^a^ Charlson index: All CCI indicators

For the socio-demographic features, we use the patient’s age, gender, disposable family income, level of education, occupation, and an indicator for whether the patient lives alone. Data on age, gender, and marital status are retrieved from the Population Register [23], and data on occupation and family disposable income is from the Income Statistics Register [24]. All socio-demographic features are measured at the point of testing. Education is measured on a 5-level scale: primary school, upper secondary, medium long, long, and unknown, where-as occupation is measured on a 6-level scale. Besides the HbA1c levels, we include clinical biomarkers test scores on cholesterol, creatinine, and triglyceride from the laboratory test database. The socio-economic and laboratory features are selected based on their theoretical association with HbA1c (behavioural as well as clinical) and availability in the data set [25].

Data on GP utilisation are gathered from The National Health Insurance Service Registry [26]. In total, we look at 36 different services and types of consultations in the six-month period prior to the test date. The most frequent services were face-to-face-consultations followed by email- and telephone consultations. The inclusion of GP utilisation is particularly relevant, as individuals with additional health issues may visit their GP more frequently, potentially impacting their diabetes management. All services are shown in the S1 Table. To measure recent diagnoses, we extract ICD-10 diagnosis codes from hospital records six months prior to the test date. To reduce possible over-fitting and sparsity problems, we use the first three digits of the ICD-10 codes and remove codes only present among 5% of the patients. Sparsity problems refers to datasets with many features with zero values. Prescription medicine is derived using level 4 of ATC codes for groups of medicines mainly used by patients with T2D. A full list of included ICD-10 codes and ATC codes are available in the S1 Table.

### The prediction model

The prediction problem is a multiclass classification problem where the target variable is based on the concept of ‘time-to-next-positive-test’. In essence, the outcomes within the training dataset are derived from the duration it takes for the tests to become positive, surpassing the threshold of 48 mmol/mol. We define the optimal test interval such that it aims at dismissing tests that are expected to be futile in the sense that its predicted value is not elevated. This means that the optimal interval is defined by the lowest interval (3, 6, 9 or 12 months) for which the predicted HbA1c level is not elevated. This rule can be expressed as follows:

$$\begin{array}{c} y=\{\begin{array}{cc} 3\;\text{months} & \text{if}\;0\leq T<183\;\text{days}\\ 6\;\text{months} & \text{if}\;183\;\text{days}\leq T<274\;\text{days}\\ 9\;\text{months} & \text{if}\;274\;\text{days}\leq T<365\;\text{days}\\ 12\;\text{months} & \text{if}\;365\;\text{days}\leq T<457\;\text{days} \end{array} \end{array}$$ (1)

Where *T* is the time to the next positive (elevated) test.

Positive tests occurring within the 0 to 183 days interval are assigned an optimal testing interval of 3 months. Similarly, observations with a window of 184 to 274 days and 275 to 365 days between positive tests are assigned to an optimal interval of 6 months and 9 months, respectively, while the 12 month interval defines the cases with no positive test results within 457 days. This also includes patients who do not experience positive tests during the study period.

The prediction model is based on the XGBoost framework. XGBoost is an advanced and efficient implementation of the gradient boosting framework, specifically designed for speed and performance. The gradient boosting framework is a ML algorithm for regression and classification problems. The algorithm constructs a prediction model in the form of an ensemble of weak prediction models, typically decision trees [27, 28]. XGBoost constitutes a gradient tree boosting approach augmented by various extensions [27].

In addition to using XGBoost model, we also run Random Forest (RF) and Logistic Regression (LR) models and compare their results with XGBoost. Since RF and LR models cannot handle missing data directly, we impute missing values for continuous variables using the median. This imputation ensures consistency and allows for a meaningful comparison of performance across all models.

In real-world scenarios, the distribution of data among different categories within a dataset are often imbalanced. This results in certain classes having a lower number of samples (referred to as the minority class), while other classes have a larger representation (known as majority classes) [29, 30]. In our study, these ‘classes’ correspond to different testing intervals. This phenomenon can lead to biased model performance, as predictive algorithms tend to favor the majority class due to their greater prevalence in the dataset. In the current study, the outcomes of interest are categorised into four distinct time frames: 3, 6, 9, and 12 months. The distribution of these outcomes within the original dataset exhibit substantial imbalance, with certain time frames being observed significantly less frequently than others. For instance, the 3-month outcome appear nearly 14 times as often as the 9-month outcome. Such disparities can lead to inadequate learning of minority classes and thus, compromised model generalisation [31]. To mitigate the challenges posed by outcome imbalance, we apply a downsampling strategy, a commonly used approach in the literature [30]. The downsampling strategy focuses on the majority class to ensure a more equitable representation of each outcome category. This approach involves randomly selecting a subset of instances from the majority class such that the number of instances in the majority class matches the size of the smallest class. Downsampling is performed only on the training data, ensuring that the test datasets remain untouched for a fair evaluation of the model’s performance. Before downsampling, the data is randomly divided into training and test data. The split is done at the individual level, meaning that all tests for an individual will either be in training or test data. The split is done such that 80% is in training and 20% in test data as recommended in the ML literature [32]. After the split, the training data were downsampled, resulting in 19,978 tests in each category.

#### Cross-validation

To further enhance the robustness of our model evaluation, we apply 5-fold cross-validation (CV). CV divides the training data into five subsets, or “folds.” In each iteration, the model is trained on four of these folds and validated on the remaining one. This process is repeated five times, implying that each fold is used as a validation set once. Using 5-fold CV ensures that the model’s performance is tested on various segments of the data, reducing the risk of overfitting and providing a more reliable estimate of its generalisation performance. By leveraging CV, we assess the model’s predictive consistency across different data subsets, resulting in a more stable and well-tuned final model. The benefits of CV for models like XGBoost and RF are well-established. It assists in hyperparameter optimisation by systematically exploring the hyperparameter space [33, 34] and improves model stability by testing performance across multiple independent folds [35, 36]. Furthermore, CV enables an unbiased comparison of models, facilitating the selection of the best approach for the task [37]. Our final XGBoost model (Table 2) has the following hyperparameters:

**Table 2:** Model information.

Objective: Multi:softprob
Evaluation metric: AUC
Eta: 0.01
Lambda: 0.9
Number of classes: 4
Gamma: 3
Max depth: 10
Min child weight: 5
Booster: gbtree
Alpha: 0.5

#### Performance evaluation

The predictive performance of the models is assessed using ROC-AUC curves. We apply a one vs one (OVO) approach when assessing the ROC-AUC to evaluate the model’s performance. This method allows us to evaluate the model by comparing each class against another. For instance, we consider one class, such as 3 months, as the “positive” class, while another class, such as 6 months, is deemed the “negative” class. By adopting this approach, we transform the multiclass classification output into a binary classification problem, allowing us to effectively leverage all established binary classification metrics to evaluate the scenario.

Besides using ROC-AUC to assess performance, we also use the confusion matrix to examine the performance by split of True Positives (TP), True Negatives (TN), False Positives (FP), and False Negatives (FN) [38]. We do this to get a deeper understanding of the models performance and to evaluate this risk of wrong predictions.

The cases that are the most clinically controversial to our prediction model are the ‘deprived’ cases where individuals are assigned to a longer test intervals than optimal resulting in unmet needs. These cases are also known as the false negatives (FN). FN can be found in the confusion matrix, and the formula is defined as follows:

$$\begin{array}{c} \text{FNR}=\frac{FN}{TP+FN} \end{array}$$ (2)

We are specifically interested in reducing the risk of FN’s in the prediction. To assess this balance, we report the false negative rates and analyse if any systematic FN classification exists.

#### Feature importance

We employ SHAP values (Shapley additive explanations) to identify the most influential features impacting predictions. The SHAP method is a visualisation technique introduced by Lundberg et al, 2017 [20]. It is based on Shapley values and is widely used for interpreting the significance of both global and local factors in ML-based prediction models. It is achieved by assessing the impact of each risk factor through SHAP values. The method is rooted in coalitional game theory, where predictors function as players within a coalition [39]. The SHAP method ensures equitable solutions for each player in the model’s outcome and adheres to a set of commendable properties such as consistency, efficiency, dummy, and additivity. Compared to alternative approaches, like local interpretable model-agnostic explanations (LIME), the efficiency characteristic of the SHAP method yields more dependable results of the contribution of each factor with estimates of the magnitude and direction (sign) of feature importance [40].

We also examine model performance using feature importance. While SHAP values provide local interpretations by showing the contribution of each feature to individual predictions at specific time points (e.g., 3 months vs. 12 months), the feature importance method provides a global view of feature relevance. Feature importance ranks the overall contribution of features to the model’s predictive performance, averaged across all outcomes (e.g., 3, 6, 9, and 12 months) [20].

As an additional test, we run the model including only the top 20 most important features identified through the feature importance plot. The goal is to evaluate whether the model can maintain or improve its performance compared to when all features are used. By focusing on a reduced set of the most impactful variables, we aim to identify any redundant features that may not significantly contribute to the model’s predictive capability. If the model performs similarly or better with just the top 20 features, this would suggest that some variables in the full model may be unnecessary, introducing noise or redundancy. This test allows us to optimise model complexity without compromising predictive accuracy.

#### Fairness

To measure fairness in the ML models, we focus on educational level and income distribution quartiles, both tied to personal barriers. It is likely that there is a educational gradient in patients’ self-management skills and utilisation of healthcare beyond need due to differences in e.g., health literacy skills [41–43].

Moreover, despite free healthcare, low-income individuals may face barriers to access due to limited work flexibility and higher travel costs, while higher-income individuals may prioritize health management to safeguard their earning capacity and career [44]. Educational backgrounds are categorised into five levels, while income brackets range from below the 10th percentile to above the 90th percentile. Predictive accuracy may vary across educational levels, particularly between those with primary school education and those with higher educational backgrounds. Instances with unknown educational data may also exhibit distinct accuracy rates. Similarly, prediction accuracy may vary among income groups, with differences observed between the lowest income group (below 10th percentile), middle-income, and the highest income group (above 90th percentile groups). To evaluate the fairness of our predictive model, particularly in instances where patients with T2D may be incorrectly assigned longer testing intervals than recommended, we utilise the false negative parity ratio (FNPR) and examine if our model maintains equitable and safe predictive abilities across diverse patient groups [45, 46]. FNPR is derived by comparing the false negative rates (FNRs) of different subgroups, providing a metric to identify any disparities in predictive performance across these distinct groups.

As standard in the ML literature, we use a range of 0.8—1.25 as threshold for determining whether our model exhibits statistically significant levels of disparity. The estimates can be interpreted as group-based unfairness that occur when a model has different outcomes or predictions for different groups of individuals, based on their membership in a protected class [47]. The FNPR quantifies incorrectly predicted negatives. A value of 1 denotes perfect parity between groups, a value below 1 indicates the unprivileged group’s lower FNR than the privileged group, and a value above 1 indicates the opposite. Values outside 0.8 to 1.25 signal significant group-based unfairness, warranting investigation and potential mitigation [48].

Our analysis prioritises evaluating fairness on the newly introduced 9 and 12-month intervals for HbA1c testing, as these are outside the standard guidelines of 3 and 6 months. This priority arises from recognising that while some individuals may manage T2D with extended testing intervals, ensuring precise and equitable predictions with these longer duration’s is essential to prevent deprivation of healthcare services for patients with an unmet need.

### Ethics

This study is based on micro-data analysed at a server on Statistics Denmark. The study complies with GDPR and Danish data security regulations. The Regional Scientific Ethical Committees for Southern Denmark assessed the study and concluded that no further ethical approval was needed (cf. section 14, subsection 1, Act on Research Ethics Review of Health Research Projects—file number: 20192000–99).

## Results

### Model performance

#### Effect of downsampling, cross-validation and model comparison

We evaluate the effect of downsampling on model performance by comparing the AUC scores of models trained on the original imbalanced dataset versus the balanced dataset created through downsampling. As shown in Table 3, downsampling impacts model performance across all models. For XGBoost, the AUC improves from 0.689 (imbalanced) to 0.732 (balanced). The RF and LR models exhibited similar trends, with AUC increasing from 0.689 to 0.728 and 0.676 to 0.680, respectively, after downsampling. Adding cross-validation further improved the models, also seen in Table 3. For the XGBoost model, the overall AUC was 0.732 without CV and 0.736 with CV, demonstrating consistent performance. Similarly, the RF model improved from 0.728 without CV to 0.733 with CV, and the LR model improved from 0.680 without CV to 0.682 with CV. These improvements demonstrate the importance of addressing class imbalance for enhancing model predictive performance. Among the three models, XGBoost consistently achieves the highest AUC, particularly on the balanced dataset, outperforming RF and LR. The superior performance of XGBoost, combined with its ability to handle missing data without preprocessing, reinforces its selection as the model of choice for this analysis. For more detailed performance metrics, including sensitivity and specificity for each model and time interval, see the S3 Table.

**Table 3:** Comparison of model performance across downsampling and cross-validation settings.

Model	AUC (Imbalanced + no CV)	AUC (Balanced + no CV)	AUC (Balanced + CV)
XGBoost	0.689	0.732	0.736
Random Forest	0.689	0.728	0.733
Logistic Regression	0.676	0.680	0.682

#### Area under the curve

**Fig 2:**
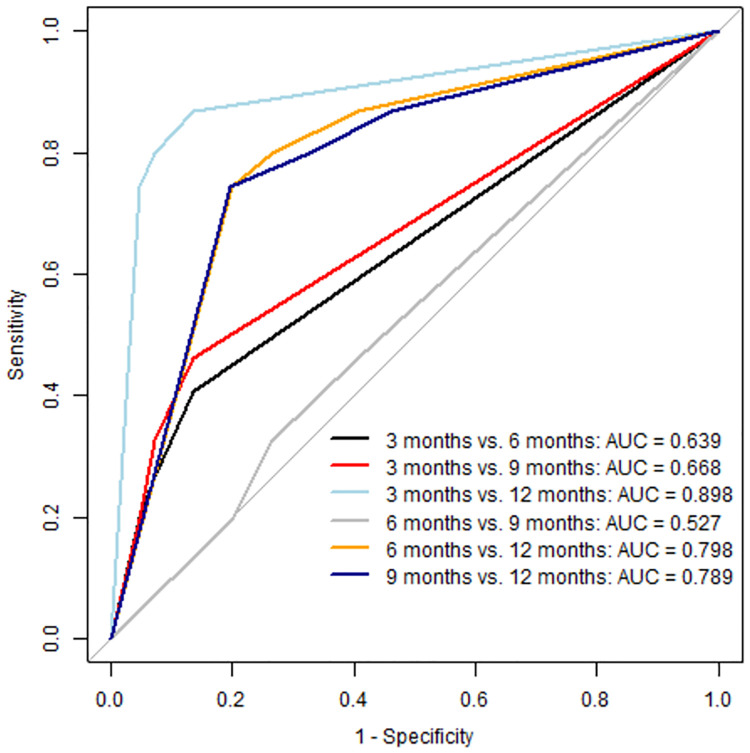
Multiclass—AUC ROC Curve for XGBoost model.

Fig 2 illustrates the Area Under the Curve (AUC) values and corresponding curves obtained using a one-versus-one approach for the four classes for the XGBoost model: 3 months, 6 months, 9 months, and 12 months. As shown in Fig 2, the AUC scores range from 0.53 to 0.89, indicating the varying effectiveness of the XGBoost model in distinguishing between different time intervals for HbA1c measurements. The lowest AUC score is observed for the comparison between 6 and 9 months (AUC = 0.53), suggesting that the model faces relatively big challenges when distinguishing between these two classes. In contrast, the highest AUC score for comparing 3 and 12 months (AUC = 0.89) indicates that the model discriminates well between these time intervals.

#### The confusion matrix

The results of the confusion matrix for XGBoost is shown in Table 4. The confusion matrix provides a comprehensive view of the model’s classification performance by illustrating the distribution of predicted versus actual classification of optimal intervals for HbA1c testing. The columns represent the predicted optimal intervals, while the rows represent the true interval to a positive test result according to the definition in Eq 1. We find that 64.3% of the predictions are given a correct interval (on the diagonal), while 22.9% are given an interval that is longer than recommended (above the diagonal), and 12.8% are given an interval that should have been longer (under the diagonal). The considerable number of correct predictions primarily stems from the 3-month and 12-month intervals, which also exhibit the highest prevalence within the dataset. Instances above the diagonal in the confusion matrix are of particular interest, as they illustrate the potential risk of miss-classifying patient to longer test intervals than optimal. This could compromise patient health and, therefore, prevent physicians from adopting the predictions. By comparing the percentage of tests that could have been assigned a longer interval (12.8%) with the proportion in the overall study population (approximately 30%), the model has effectively reduced the incidence of over-provision of tests.

**Table 4:** Confusion matrix—XGBoost.

Predicted optimal interval					
		3 months	6 months	9 months	12 months
True interval between elevated tests	3 months	**39250**	10260	4012	3337
	6 months	2967	**2383**	1103	1820
	9 months	1018	1180	**1004**	1366
	12 months	2135	3396	1599	**18992**

#### Features importance

**Fig 3:**
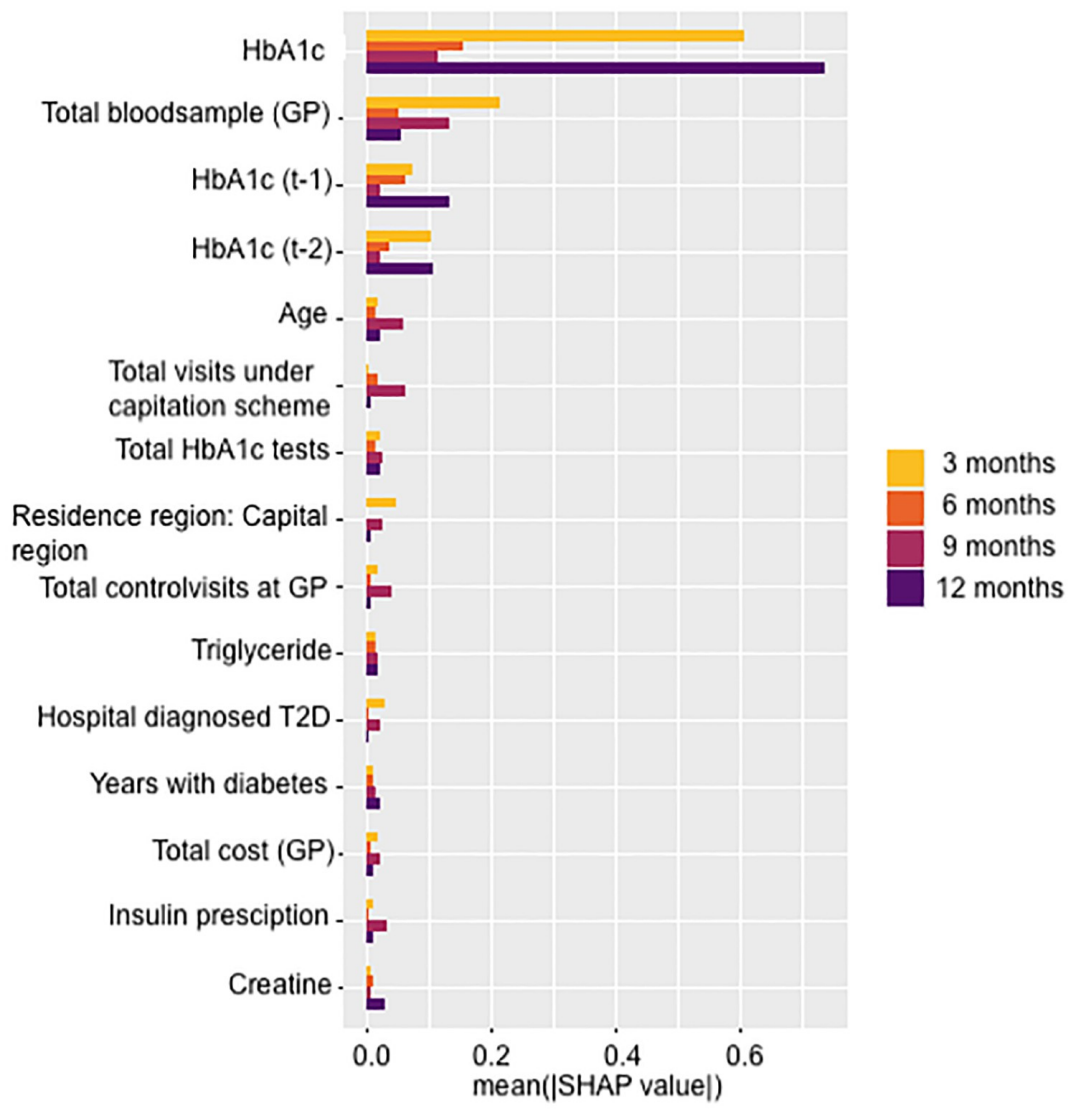
SHAP.

The visual analysis reveals central patterns in mean SHAP values Fig 3. HbA1c consistently holds the highest mean SHAP value across all outcomes, with notably heightened influence at 3 and 12 months compared to 6 and 9 months. “Total blood samples at the GP” follow closely, showing substantial impact at 3 and 9 months. HbA1c in t-1 and t-2 rank third and fourth. Notably, “Age” and “Total visits under capitation scheme” show heightened influence for the 9-month outcome, differentiating their impact across intervals.

**Fig 4:**
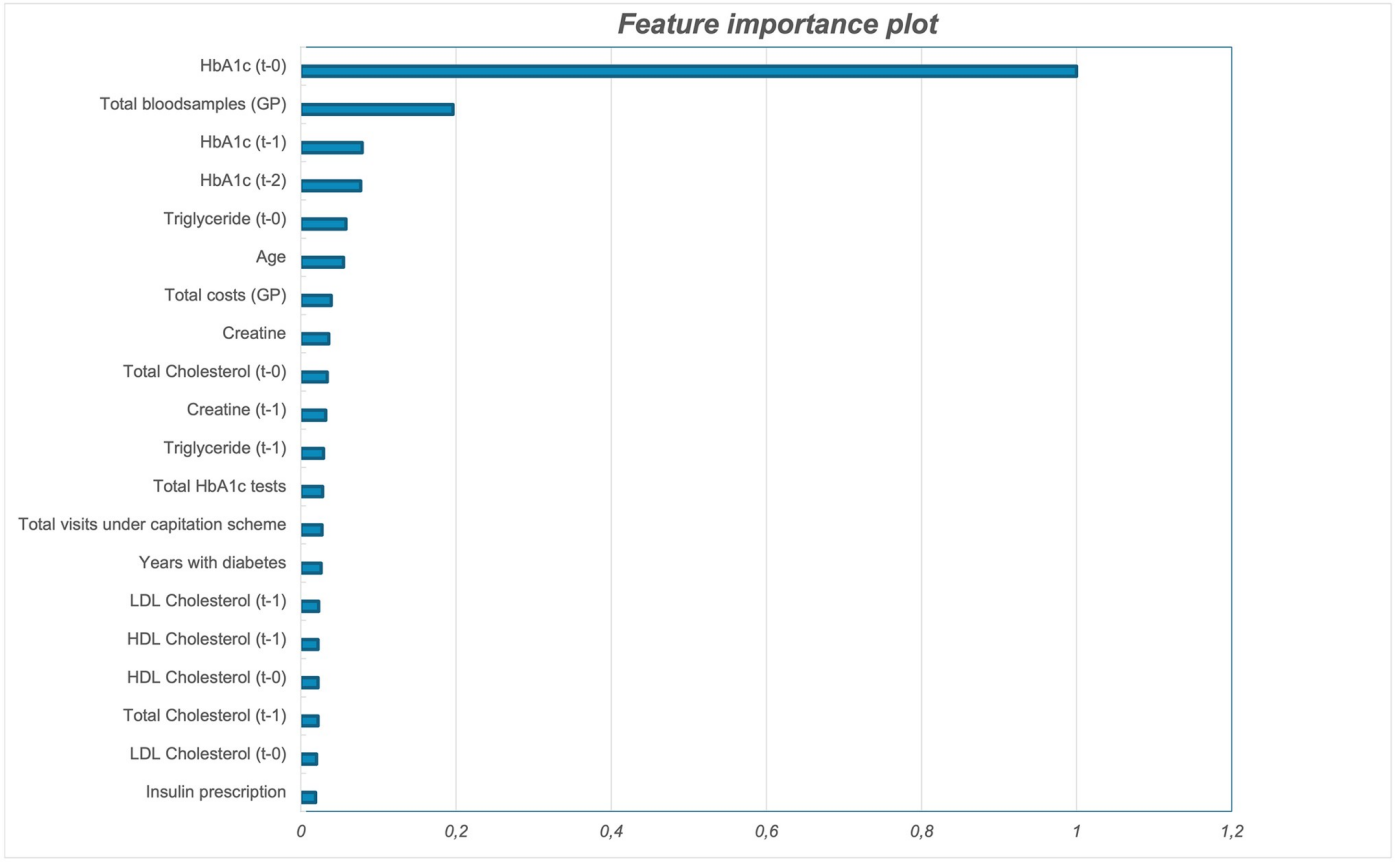
Feature importance.

A comparison between Fig 3 (SHAP values) and Fig 4 (feature importance) shows that the identified key features are largely consistent across methods, with only slight variations in rankings. To evaluate the impact of feature selection, we also run the model only including the top 20 most important features. The reduced model obtain an AUC score comparable to our base model with all features (see S5 Table), suggesting that many of the less important features may be redundant.

#### Fairness

The figures below show the measures for fairness in the ML model by education and income. We focus on assessing fairness regarding the newly introduced longer 9 and 12-month intervals for HbA1c testing, which deviate from the standard guidelines of 3 and 6 months. While some individuals may manage T2D with extended testing intervals, it’s crucial to ensure accurate and equitable predictions for implementation of longer duration’s to prevent deprived access to healthcare services for those in need irrespective of socio-economic position.

**Fig 5:**
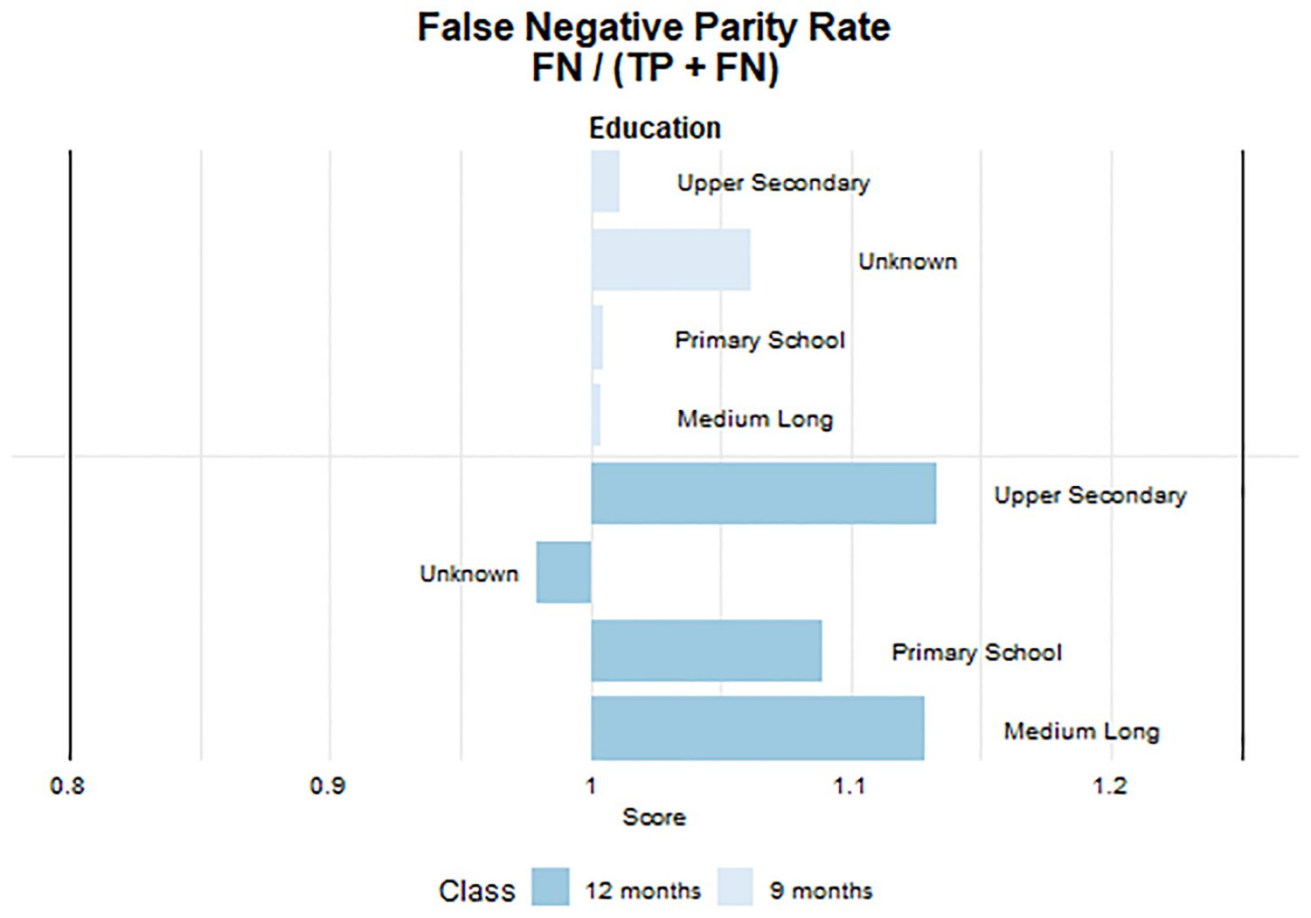
Fairness—Education Fairness is indicated when the bar falls within the range of 0.8 to 1.25.

Fig 5 provides detailed insight into the fairness metrics “False Negative Rate Parity,” across different levels of education. This metric is integral to understanding the potential biases that may arise in predictive models across educational level. The figure shows that none of the groups surpass or fall below the unfairness threshold, demonstrating that the ML model is unbiased across education levels. Nevertheless, we do some variation in the risk of receiving false negative predictions across the education groups. For instance, the group with medium education demonstrates a higher likelihood of false negative predictions compared to the group with higher education for the 12 months interval.

**Fig 6:**
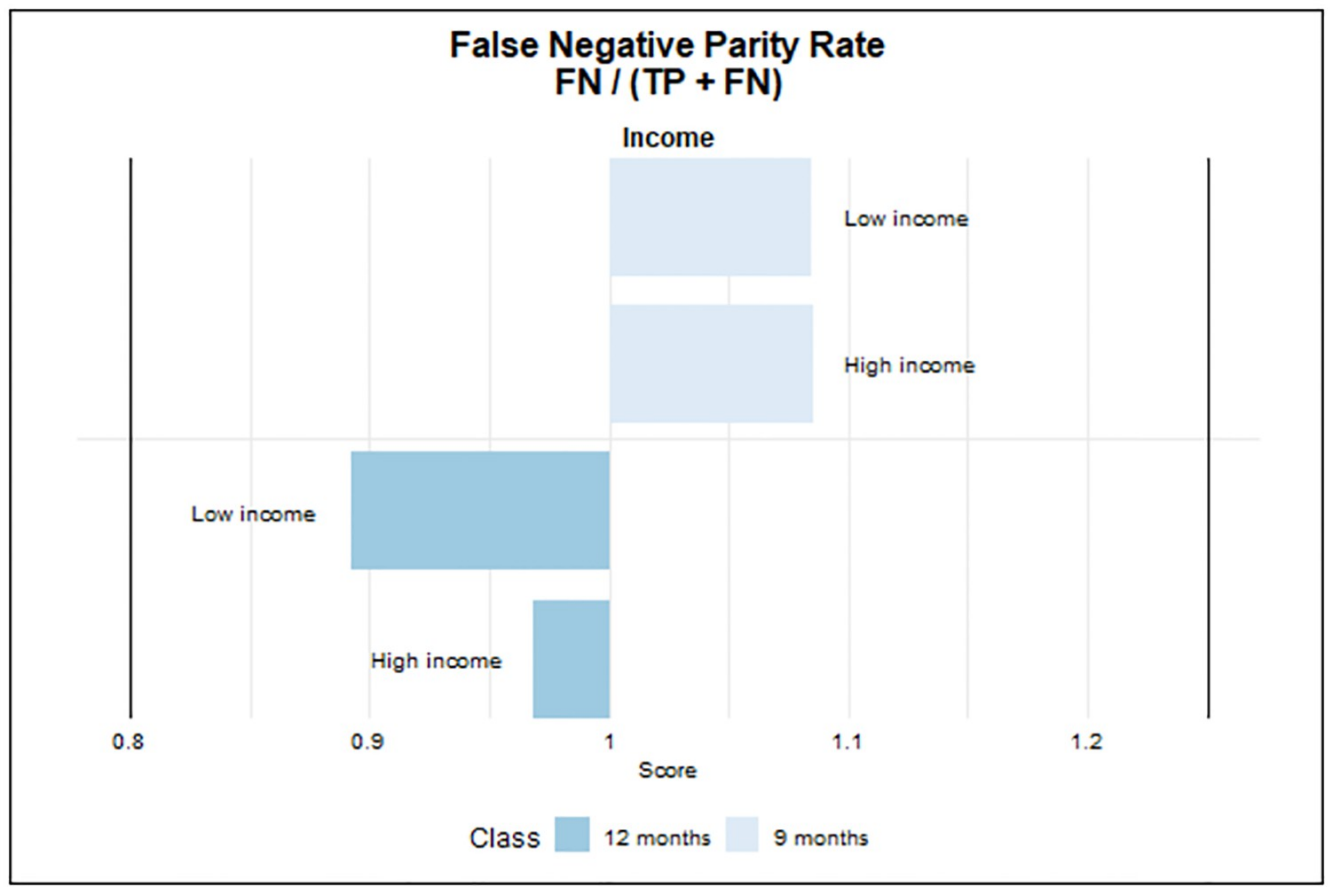
Fairness—Income Fairness is indicated when the bar falls within the range of 0.8 to 1.25.

Fig 6 reveals that there is no detectable unfairness across income brackets indicating that the ML model treats income groups fairly. However, both low- and high-income groups appear to have a reduced risk of receiving false negative predictions for the 12-month interval compared to the medium income group.

## Discussion

The present paper demonstrates the potential of using ML to guide personalised healthcare in the monitoring of chronic patient groups. Our model provides effective predictions of implementing longer test intervals for well-controlled T2D patients and shorter intervals for patients who struggle to maintain blood glucose levels below the recommended level. The most important features are previous HbA1c levels, total blood samples taken at the GP and the patient’s age. Information that in most healthcare systems should be accessible to the healthcare provider.

Despite challenges to distinguish between 6 and 9-month intervals (AUC = 0.53), the XGBoost model effectively discriminated between 3 and 12-month intervals (AUC = 0.89). This disparity may be attributed to the imbalance in our out-of-sample data, which predominantly comprises tests requiring intervals of 3 and 12 months. Achieving a more balanced dataset could potentially enhance the model’s performance at 6- and 9-month intervals. Whereas 64.3% of predictions are correct, the model resulted in wrong classification in 22.9% (longer interval than optimal) and 12.8% (shorter interval than optimal) of the predictions. Comparing the 12.8% to the current test practice in the sampled population (where around 30% of the tests are below the critical value), the application of our model has the potential to reduce futile testing and thereby free resources to be allocated to T2D patients in need of more support. While this result is promising, it comes at a cost for the 23% cases that will incorrectly receive less support, resulting in longer intervals between test monitoring and an increased risk of prolonged periods with elevated glucose levels. We initially evaluated three models and decided to continue with the XGBoost model. While the Random Forest algorithm performs equally well to XGBoost, it is limited in its ability to handle missing data directly, requiring imputation steps that can add complexity and potential bias. In contrast, XGBoost natively handles missing data, which simplifies the modelling process and reduces the risk of errors introduced by imputation. Moreover, XGBoost generally offers faster training times and better scalability with large datasets, making it a more efficient choice for our analysis.

Our findings indicate that prediction models relying on Danish register data may be less prone to unfairness. Reassuringly, and in contrast to other studies [49], we find an indication of fairness in model predictions across education and income groups. In Denmark, healthcare is universally accessible regardless of socio-economic status, with free access to primary and secondary care, which is likely to reduce the socio-economic barriers to access to healthcare. Moreover, to reduce potential selection bias and thus unfairness in the health data, we restrain our prediction problem to ‘regular users’, i.e., individuals with T2D who use healthcare and who are regularly monitored at the GP. By doing this, we seek to ensure that all eligible patients are well-represented in our data, mitigating the risk of selection bias in the health data.

The applicability of our prediction model depends on the assumptions that the frequency of testing does not directly affect HbA1c levels and that patients who are tested less frequently do not differ in their diabetes management abilities compared to those who are tested more frequently. If these assumptions are not met, it could undermine the validity of the model’s conclusions. Additional research is needed to examine if adopting a less frequent testing strategy can influence patient health behaviour and outcome and affect predictive performance. We assert that ML-guided personalised medicine should be followed by careful assessments of potential effects on the underlying causal questions. In the present study, this involves recognising that identifying different frequencies of HbA1c monitoring aimed at preventing disease progression presents a challenging causal inference problem that requires estimating individual treatment effects of various frequency models. Health systems often rely on one-size-fits-all program guidelines based on clinical or biological reasoning. For example, the frequency of HbA1c testing is often guided by the time it takes blood cells to recover rather than the specific needs of individual patients [50]. However, assuming that the HbA1c level is independent of the time interval between tests, we can simplify the causal problem into a prediction problem [11]: If we can accurately predict the optimal interval for the next HbA1c test well in advance of the next appointment, we can determine whether the test can be postponed for longer than the current guidelines recommend, saving healthcare costs and patient time. Nonetheless, a critical observation here is that the assumption of ‘no behavioural change’ is fundamental. What if the testing interval, irrespective of the level of HbA1c, has a direct effect on patients’ disease management and their adherence to a healthy lifestyle? The answer to this question necessitates a causal investigation and call for close monitoring of ML-guided personalised medicine.

The absence of external data is a limitation of this study, hindering our ability to evaluate the model’s robustness and stability under varying conditions. While the current study demonstrates the model’s efficacy using population data from Danish health registers, its generalisability may be limited when applied to other datasets or different clinical environments. Factors such as population heterogeneity and variations in healthcare delivery practices can influence the model’s predictive accuracy. To assess the generalisability of our findings, we apply cross-validation. Reassuringly, we find that our model performs well on unseen data, reducing the likelihood of overfitting or underfitting and enhancing the reliability of our findings, thereby increasing its potential for broader application. Future research should prioritise obtaining external dataset to rigorously assess the model’s generalisation capabilities. While our findings provide valuable insights into the potential of ML for personalised diabetes management, addressing these limitations and pursuing further research will enhance the model’s reliability and inform more effective healthcare practices.

## Conclusion

We demonstrate the potential of applying ML to support personalised healthcare delivery, with a focus on results and implementation-relevant biases. We develop a ML model to predict tailored testing interval of blood glucose for individuals with T2D and rigorously analyse the model performance and risks associated with biases, false-negative rates, and model unfairness. These results are important from an implementation perspective, acknowledging that the risk of wrong classifications could prevent physicians from pursuing personalised medicine do to concern of harming the patients. We show that our ML model can be used to predict the optimal interval for blood glucose testing using available administrative and clinical health data. We find that almost one third of the tests among the T2D patients who are adherent to the current “one size fits all” testing frequency can be giving a longer time interval. While the model performs well in terms of fairness and refrains from augmenting socio-economic inequalities in access, it incorrectly predicts longer test intervals in 1 out of 5 cases, which is a concern. We believe that the findings provides important insight to the field of diabetes management and resource optimisation of healthcare for chronic patients. The utilisation of ML models to predict personalised optimal testing intervals for HbA1c in patients with T2D holds the potential to reshape clinical practices and healthcare policies in several ways. Firstly, implementation for ML models has the potential to free healthcare resources by minimising the frequency of HbA1c tests for patients who do not require frequent monitoring. Secondly, it provides a patient-centered approach to diabetes monitoring that has the potential to improve equity in health by reallocating resources according to need. Yet, in order to implement the model in clinical practice, the model needs to undergo some more testing especially clinical validation on diverse patient populations to assess its performance in real-world settings.

## Supporting information

S1 Table Variables included in the analysis. (PDF)

S2 Table Descriptive between test data and training data. (PDF)

S3 Table Performance of the models with and without downsampling. (PDF)

S4 Table Confusion matrix for XGBoost with only top 20 features. (PDF)

S5 Table Performance of XGBoost with only top 20 features. (PDF)
