# Supplementary material for: Optimising test intervals for individuals with type 2 diabetes: A machine learning approach
Source: PLoS One. 2025 Feb 13;20(2):e0317722. doi: 10.1371/journal.pone.0317722 (PMC11824975; doi:10.1371/journal.pone.0317722)
Supplement: S1 Table — (PDF) [file pone.0317722.s001.pdf]

S1 Table (1) Variables included in the analysis

IDC10 diagnosis	E11, E78, H25, H91, I10, i25, I48, R06, Z01, Z03, Z09, Z50, Z95
Medication utilization:	ATC_A02B, ATC_A06A, ATC_A10A, ATC_A10B, ATC_A12B, ATC_B01A, ATC_B03B, ATC_C01D, ATC_C03A, ATC_C03C, ATC_C03D, ATC_C05A, ATC_C07A, ATC_C08C, ATC_C09A, ATC_C09B, ATC_C09C, ATC_C09D, ATC_C10A, ATC_D01A, ATC_D06A, ATC_D07A, ATC_G03C, ATC_G04B, ATC_G04C, ATC_H02A, ATC_H03A, ATC_J01C, ATC_J01E, ATC_J01F, ATC_J01M, ATC_J02A, ATC_M01A, ATC_M04A, ATC_N02A, ATC_N02B, ATC_N03A, ATC_N05A, ATC_N05B, ATC_N06A, ATC_R01A, ATC_R03A, ATC_R03B, ATC_R05D, ATC_R06A, ATC_S01A, ATC_S01B, ATC_S01C, ATC_S01E
Test at the GP	Total EKG, total CRP, total bloodsample, total urine stix, total urine microscopy, total urine culture, total culture bio, total strep A, total hemoglobin, total spirometry ord, total spirometry ext, total hbac, total glucose,
GP contacts	Total consultation, total email, total telephone, total home visits, total home visit prev, total controlvisits, total consultation diab, total email diab, total telephone diab, total cap service, total cap copd, total cap diab, total cap copd diab, total cap stop, total influenza, total hearing test, total minor surgery, total near patient, total contracep pill, total contracep implant, total contracept consult, total labatory test
