# Supplementary material for: Optimising test intervals for individuals with type 2 diabetes: A machine learning approach
Source: PLoS One. 2025 Feb 13;20(2):e0317722. doi: 10.1371/journal.pone.0317722 (PMC11824975; doi:10.1371/journal.pone.0317722)
Supplement: S2 Table — (PDF) [file pone.0317722.s002.pdf]

S1 Table (2) Descriptive between test data and training data

Category	Test data (N=95822)	Training data (N=79912)	P-value
**Sex**			
Male	54280 (57 %)	46038 (58 %)	<0.01
**Age (years)**			
Mean (SD)	59.29 (± 7.761)	58.85 (± 8.298)	<0.001
**Single (living alone)**			
Single	27431 (29 %)	23448 (29 %)	<0.001
**Ethnicity**			
Non-danish	14327 (15 %)	13728 (17 %)	<0.001
**Education**			
Primary School	35865 (37 %)	29159 (36 %)	<0.001
Upper Secondary	45104 (47 %)	37926 (47 %)	
Medium Long	9828 (10 %)	8026 (10 %)	
Long	2373 (2 %)	2550 (3 %)	
Missing	2652 (3 %)	2251 (3 %)	
**Occupation**			
Employed	34102 (36 %)	29375 (37 %)	<0.001
Unemployed	8932 (9 %)	7935 (10 %)	
Education	179 (0 %)	179 (0 %)	
Retired	50807 (53 %)	40867 (51 %)	
Other	1802 (2 %)	1556 (2 %)	
**Region**			
North Jutland Region	22274 (23 %)	14007 (18 %)	<0.001
Region of Southern Denmark	23363 (24 %)	21895 (27 %)	
Capital Region	19634 (20 %)	22148 (28 %)	
Region Zealand	30551 (32 %)	21862 (27 %)	
**Family income (DKK)**			
Mean (SD)	243400 (± 131800)	248100 (± 229800)	0.537
Missing	63 (0.1%)	24 (0.0%)	
**Years with diabetes**			
Under 5 years	49533 (52 %)	37449 (47 %)	<0.001
Above 5 years	46289 (48 %)	42463 (53 %)	
**Age at diabetes diagnosis**			
Mean (SD)	50.90 (± 8.571)	50.12 (± 9.363)	<0.001
**HbA1c (mmol/mol)**			
Mean (SD)	54.76 (± 12.85)	55.14 (± 13.97)	<0.001
**HbA1c t-1 (mmol/mol)**			
Mean (SD)	54.64 (± 12.95)	55.34 (± 14.35)	<0.001
**HbA1c t-2 (mmol/mol)**			
Mean (SD)	54.37 (± 12.85)	54.79 (± 14.06)	<0.001
Missing	4745 (5.0%)	9330 (11.7%)	
**LDL cholesterol t-0 (mmol/l)**			
Mean (SD)	2.025 (± 0.8412)	2.069 (± 0.8573)	<0.001
Missing	9253 (9.7%)	12751 (16.0%)	
**LDL cholesterol t-1 (mmol/l)**			
Mean (SD)	2.041 (± 0.8459)	2.090 (± 0.8644)	<0.001
Missing	11225 (11.7%)	15490 (19.4%)	
**HDL cholesterol t-0 (mmol/l)**			
Mean (SD)	1.227 (± 0.3601)	1.229 (± 0.3708)	0.135
Missing	3920 (4.1%)	6934 (8.7%)	
**Total cholesterol t-0 (mmol/l)**			
Mean (SD)	4.159 (± 0.9792)	4.205 (± 1.010)	<0.001
Missing	3748 (3.9%)	6722 (8.4%)	
**Total cholesterol t-1 (mmol/l)**			
Mean (SD)	4.178 (± 0.9848)	4.229 (± 1.020)	<0.001
Missing	5207 (5.4%)	8614 (10.8%)	
**Triglyceride t-0 (mmol/l)**			
Mean (SD)	2.151 (± 1.471)	2.161 (± 1.617)	0.198
Missing	3924 (4.1%)	6940 (8.7%)	
**Triglyceride t-1 (mmol/l)**			
Mean (SD)	2.150 (± 1.477)	2.169 (± 1.663)	0.012
Missing	5420 (5.7%)	8859 (11.1%)	

Continued from previous page			
**Category**	**Test data (N=95822)**	**Training data (N=79912)**	**P-value**
**Creatinine t-0 (micromole/l)**			
Mean (SD)	75.18 (± 23.17)	76.19 (± 25.59)	<0.001
Missing	15304 (16.0%)	17381 (21.8%)	
**Creatinine t-1 (micromole/l)**			
Mean (SD)	75.45 (± 23.95)	75.63 (± 25.14)	0.165
Missing	14375 (15.0%)	15432 (21.4%)	
**Charlson Comorbidity score (CCI)**			
Mean (SD)	0.3398 (± 0.8577)	0.3789 (± 0.8866)	<0.001
**Total cost (GP)**			
Mean (SD)	2654 (± 1508)	2388 (± 1426)	<0.001
**Total consultation (GP)**			
Mean (SD)	9.706 (± 6.306)	8.667 (± 6.022)	<0.001
**Total email (GP)**			
Mean (SD)	2.417 (± 4.700)	2.268 (± 4.599)	<0.001
**Total telephone contacts (GP)**			
Mean (SD)	4.641 (± 6.514)	4.431 (± 5.879)	0.803
