# Supplementary material for: Optimising test intervals for individuals with type 2 diabetes: A machine learning approach
Source: PLoS One. 2025 Feb 13;20(2):e0317722. doi: 10.1371/journal.pone.0317722 (PMC11824975; doi:10.1371/journal.pone.0317722)
Supplement: S3 Table — (PDF) [file pone.0317722.s003.pdf]

S1 Table (3) Comparison of model performance before (imbalanced) (1st) and after (balanced) applying downsampling (2nd) and with downsampling and CV (3rd). Sensitivity, specificity, and AUC values are presented for each model and time interval.

**Metric**	**Time Interval**	**XGBoost**	**Random Forest**	**Logistic Regression**
Sensitivity	3	0.788 / 0.862 /0.865	0.921 / 0.686 / 0.666	0.862 / 0.585 / 0.579
	6	0.000 / 0.136 / 0.138	0.000 / 0.237 / 0.281	0.000 / 0.099 / 0.092
	9	0.257 / 0.125 / 0.130	0.011 / 0.215 / 0.227	0.060 / 0.207 / 0.197
	12	0.700 / 0.742 / 0.744	0.784 / 0.755 / 0.726	0.756 / 0.818 / 0.829
Specificity	3	0.847 / 0.657 / 0.651	0.644 / 0.837 / 0.852	0.649 / 0.832 / 0.840
	6	0.914 / 0.926 / 0.925	1.00 / 0.856 / 0.828	0.998 / 0.936 / 0.922
	9	0.952 / 0.959 / 0.960	0.990 / 0.922 / 0.911	0.974 / 0.900 / 0.899
	12	0.914 / 0.896 / 0.899	0.875 / 0.886 / 0.907	0.849 / 0.755 / 0.087
AUC	Overall	0.689 / 0.732 / 0.736	0.689 / 0.728 / 0.733	0.676 / 0.680 / 0.682
