# Supplementary material for: Optimising test intervals for individuals with type 2 diabetes: A machine learning approach
Source: PLoS One. 2025 Feb 13;20(2):e0317722. doi: 10.1371/journal.pone.0317722 (PMC11824975; doi:10.1371/journal.pone.0317722)
Supplement: S4 Table — (PDF) [file pone.0317722.s004.pdf]

S1 Table (4) Confusion matrix - Top 20 features for XGBoost

		Predicted optimal interval			
		3 months	6 months	9 months	12 months
True interval	3 months	**38997**	10456	4069	3337
between elevated tests	6 months	2962	**2462**	1029	1820
	9 months	1056	1174	**969**	1369
	12 months	2126	3501	1493	**19002**
