# Supplementary material for: Optimising test intervals for individuals with type 2 diabetes: A machine learning approach
Source: PLoS One. 2025 Feb 13;20(2):e0317722. doi: 10.1371/journal.pone.0317722 (PMC11824975; doi:10.1371/journal.pone.0317722)
Supplement: S5 Table — (PDF) [file pone.0317722.s005.pdf]

S1 Table (5) Performance with top 20 features - XGBoost

	3 months	6 months	9 months	12 months	Overall
Sensitivity	0.8639	0.1399	0.1282	0.7444	
Specificity	0.6476	0.9257	0.9592	0.8987	
Pos Pred Value	0.6859	0.2976	0.2121	0.7274	
Neg Pred Value	0.8423	0.8272	0.9278	0.9064	
Precision	0.6859	0.2976	02121	0.7274	
Recall	0.8639	0.1399	0.1282	0.7444	
F1	0.7646	0.1904	0.1598	0.7358	
Balanced Accuracy	0.7557	0.5328	0.5437	0.8215	
Area Under the Curve (AUC)					0.734
